# A Rare Root Canal Configuration of Maxillary Second Molar: A Case Report

**DOI:** 10.1155/2012/767582

**Published:** 2012-07-08

**Authors:** Gautam P. Badole, Rakesh N. Bahadure, M. M. Warhadpande, Rajesh Kubde

**Affiliations:** ^1^Department of Conservative Dentistry and Endodontics, VSPM's Dental College and Research Center, Nagpur 440019, India; ^2^Department of Pedodontics and Preventive Dentistry, Sharad Pawar Dental College, Sawangi, Wardha 442004, India; ^3^Department of Conservative Dentistry and Endodontics, Government Dental College and Hospital, Nagpur 440009, India

## Abstract

A thorough knowledge of root canal morphology is a prerequisite for the endodontic therapy. The maxillary molars, especially the second molars, have the most complicated root canal system in permanent dentition. There are many variations in canal number and configuration in maxillary molars. Treatment may be unsuccessful because the dentist may fail to recognize the unusual canal configuration. The present paper describes a case of a right maxillary second molar with a canal configuration rarely reported in the literature. The tooth had four roots with four root canals, two individual palatal roots (mesiopalatal and distopalatal) with their own separate canals. The mesiobuccal and distobuccal root had normal anatomy. This paper may intensify the complexity of maxillary molar variation and is intended to reinforce clinician's awareness of the rare morphology of root canals.

## 1. Introduction

 The main objective of root canal treatment is to relieve pain, disinfect the root canal, and prevent reinfection [1]. To achieve clean, disinfected, and 3-dimensionally obturated root canal systems, clear knowledge of the root morphology and canal anatomy is essential. Many of the challenges faced during root canal treatment may be directly attributed to an inadequate understanding of the canal morphology of teeth. Undetected extra roots or root canals are a major reason for failure of root canal treatment [2]. Unusual canal anatomy associated with the maxillary molars has been investigated in several studies [3, 4]. Most papers have focused on the morphology of the mesiobuccal root and particularly on its mesiopalatal canal [5, 6]. However, Christie et al. [7] have reported a variation in the number of roots and an unusual morphology of root canal systems in maxillary molars. An accurate radiographic technique and proper interpretation are essential for sound diagnosis and treatment. The use of preoperative radiographs at different angles helps to detect and evaluate root canal morphology and anatomy [8].

## 2. A Case Report

**Figure 1:**
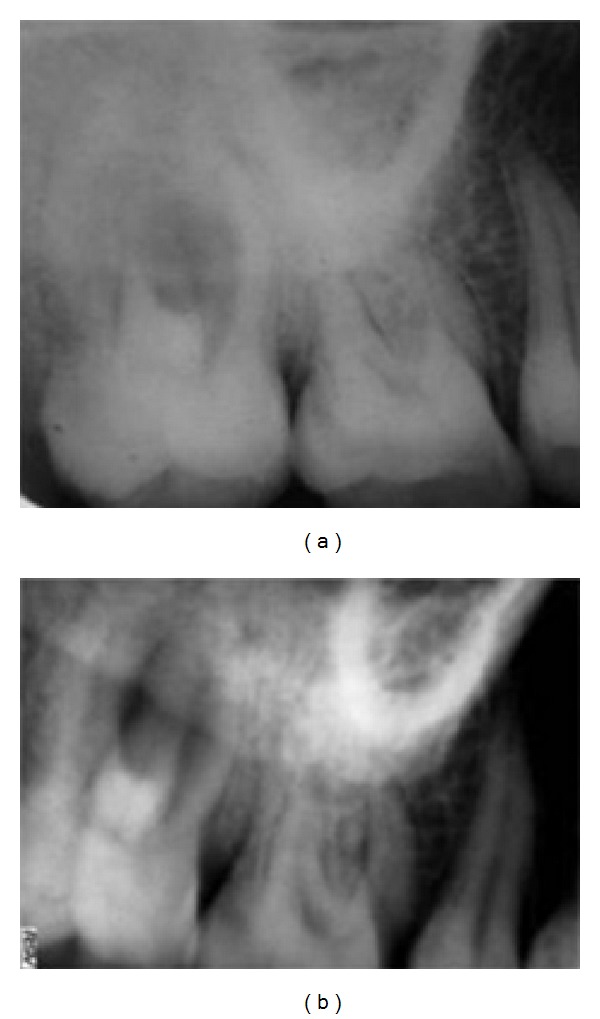
Preoperative radiograph with different angulations: complex root anatomy of maxillary right second molar.

**Figure 2:**
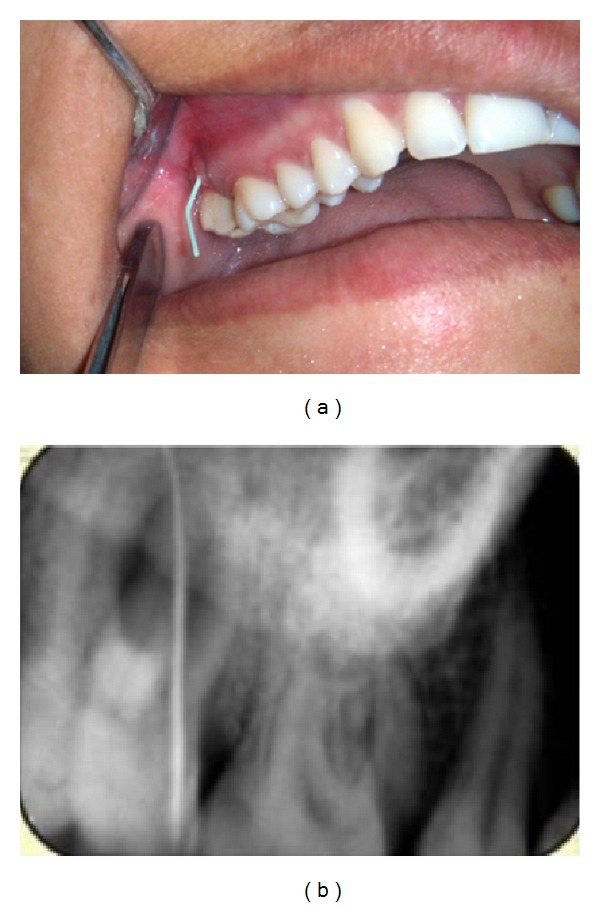
Clinical and radiographic tracing of sinus.

 A 33-year-old woman was referred in the Department of Conservative Dentistry and Endodontics for a swelling in buccal gingiva in relation to the right maxillary second molar. Clinical examination revealed the absence of caries or any other pathology with maxillary molars. The patient gave history of trauma one year back. Pain on percussion was present with the maxillary second molar. Gingival sinus tract was present on buccal-attached gingiva in relation with right maxillary second molar. A diagnostic radiograph revealed complex root anatomy. Response to vitality test was negative. Asymptomatic chronic apical periodontitis was diagnosed with maxillary second molar. Careful examination of the radiographs revealed the possibility of more than 1 palatal root (Figures 1(a) and 1(b)). Tracing of the sinus tract was done by gutta-percha (Dentsply Maillefer, Ballaigues, Switzerland) (Figures 2(a) and 2(b)). The medical history was noncontributory.

**Figure 3:**
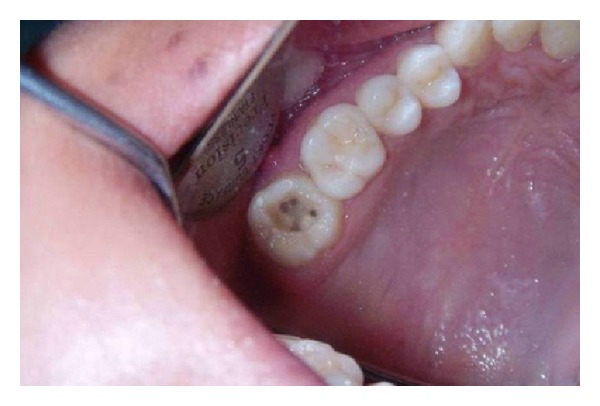
Clinical examination showing 4 root canal orifices: 2 located buccally and 2 palatally (mirror image).

**Figure 4:**
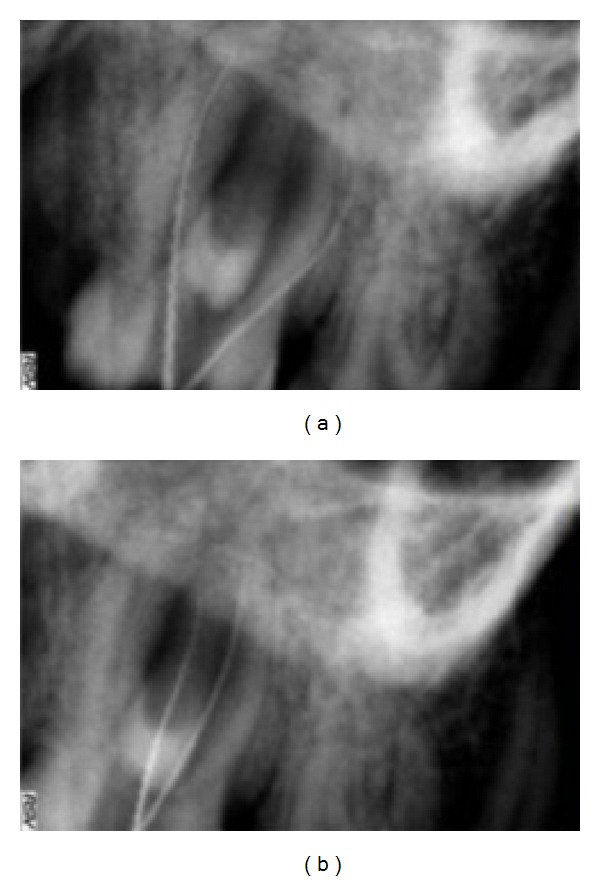
Working length radiograph of different canals.

 The tooth was anesthetized, and access to the pulp chamber was achieved using a round diamond bur (no. 4; MANI Inc., Tochigi-ken, Japan). Clinical evaluation of chamber floor confirmed the presence of 4 root canal orifices, 2 located buccally and 2 palatally (Figure 3). Working length of each canal was estimated by means of an apex locator (Root ZX: Morita, Tokyo, Japan) and confirmed with digital intraoral periapical (IOPA) radiography (Figures 4(a) and 4(b)). The root canals were cleaned and shaped using Niti rotary protapers (Dentsply Maillefer, Ballaigues, Switzerland) with crown-down technique [9]. Apical preparations in the buccal canals were enlarged to a protaper number F1, and in the palatal canals to the number of F2. The root canals were frequently irrigated with 5.25% sodium hypochlorite (Prime Dental Products, Thane, India). Calcium hydroxide (RC Cal; Prime Dental Products) paste was then placed as an intracanal medicament. The patient was recalled after 1 week, and the tooth was asymptomatic.

**Figure 5:**
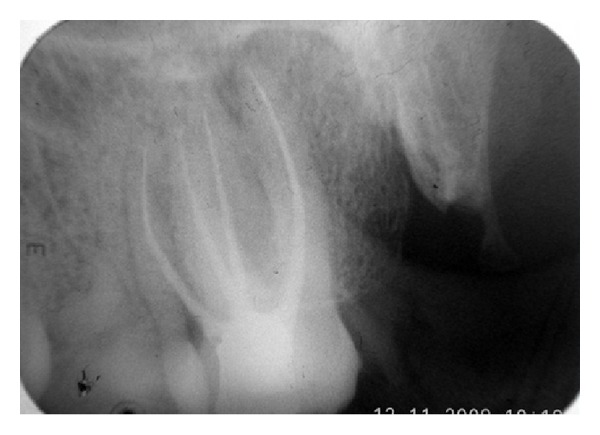
Postoperative radiograph showing the separation and divergence of the 4-rooted maxillary second molar.

**Figure 6:**
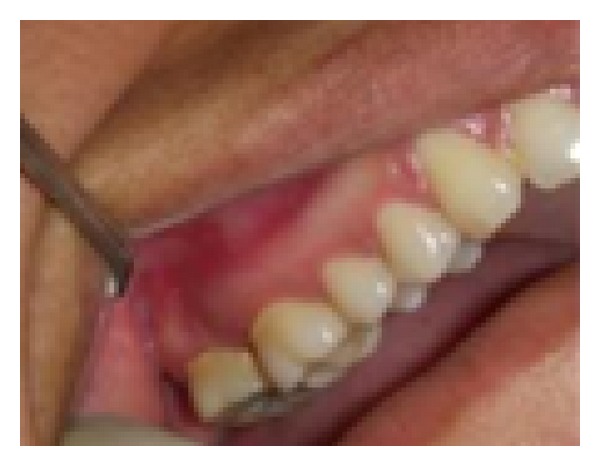
Healing of draining sinus.

 At the next appointment, the root canals were irrigated with 5.25% sodium hypochlorite and dried with paper points. Then the canals were obturated with AH-Plus sealer (Dentsply DeTrey, Konstanz, Germany) and protaper gutta-percha. After placing a silver amalgam permanent restoration, a postoperative radiograph showed the unique palatal morphology (Figure 5). One month recall of patient showed healing of draining sinus (Figure 6), and a porcelain-fused-to-metal crown was given.

## 3. Discussion

 Usually the maxillary second molars have 3 roots with 3 or 4 root canals [9, 10]. Peikoff et al. [11] demonstrated that 3.1% of maxillary second molars had 1 root and 1 canal, 2-rooted maxillary molars range from 0 to 12% [6, 10]. The prevalence of maxillary second molars with 4 roots (2 buccal and 2 palatal) is rare; it is only 0.4% [12]. Al Shalabi et al. [13] and Çalişkan et al. [14] in their *ex vivo* study found 1.2% and 3.23% two palatal foramens in maxillary second molar, respectively.

 In certain circumstances, most of the extra root canals may be left untreated during endodontic therapy [15]. Routine intraoral radiograph with different angulations helps in determination of the presence of extra roots, as demonstrated in the present case. However, diagnosis of extra canals with a normal number of roots may be difficult because of their superposition over other root canals or, sometimes, their relatively small size. Careful examination of the preoperative radiograph will aid in the detection of extra canals. Knowledge of anatomic aberrations, such as root position, root shape, and relative root outline, will also help decrease the failure rate of root canal therapy.

 Christie and others [7] had classified 4-rooted maxillary molar in 3 types on root separation level and divergence. Type I maxillary molars have two widely divergent, long, and tortuous palatal roots. The buccal roots are often “cow-horn-” shaped and less divergent. Type II maxillary molar has four separate roots, but the roots are often shorter, run parallel, and have buccal and palatal root morphology with blunt root apices. Type III maxillary molar is also constricted in root morphology with the mesiobuccal, mesiopalatal, and distopalatal canal encaged in the web of root dentin. The distobuccal root in these cases appears to stand alone and may even diverge to the distobuccal. Based on this classification, the maxillary right second molar (Figures 1 and 4) presented here could be considered a type I molar (well-separated roots).

 Properly designed and prepared access cavities is the initial step in locating canal orifices which will eliminate many potential problems during canal preparation and obturation. In case of the present paper, a large access was required on palatal side to locate the 2 palatal roots. Teeth with 2 palatal roots often have a wider mesiodistal dimension of the palatal cusps [7]. The observation of a palatogingival groove on palatal surface of crown and root indicates the chances of two palatal canals [16]. The access outline will be square rather than triangular in such cases. Clinical photograph of floor shows two well-separated palatal orifices (Figure 3). Vertucci studied the proximity of canal orifices and their separation at apical area. If the separation of orifices is greater than 3 mm, canals remain separated through the entire length and usually joined when distance is less than 3 mm [17].

 Treatment prognosis for molars with 4 canals and 2 palatal roots should be considered the same as that for any maxillary molar. Failure to treat a missed canal is an obvious reason for root canal treatment failure. Sometimes interpretation of morphologic variations on radiograph is difficult, so the visualization of pulp chamber, use of operating microscope, and electronic apex locator are more important. Recently, the use of cone beam or spiral computed tomography scan is valuable in diagnosis of anatomic variations [18–21]. Use of this aid facilitates easy detection of variations and number of root canal as compared to previously available techniques [19, 21]. Therefore, all practitioners must make every effort to locate and treat all existing canals during root canal treatment.

## 4. Conclusions

 Anatomic variation in the number of roots and root canals can occur in any tooth. Although such cases occur infrequently, dentists should be aware of them when considering endodontic treatment. Examination of clear radiographs taken from different angles and careful evaluation of the internal anatomy of teeth are essential for successful treatment. Root canal treatment is likely to fail if extra roots or root canals are not detected.
